# Plasma Treatment Limits Human Melanoma Spheroid Growth and Metastasis Independent of the Ambient Gas Composition

**DOI:** 10.3390/cancers12092570

**Published:** 2020-09-09

**Authors:** Sybille Hasse, Tita Meder, Eric Freund, Thomas von Woedtke, Sander Bekeschus

**Affiliations:** 1 ZIK *plasmatis*, Leibniz Institute for Plasma Science and Technology, Felix-Hausdorff-Str. 2, 17489 Greifswald, Germany; sybille.hasse@inp-greifswald.de (S.H.); tita-meder-@gmx.de (T.M.); eric.freund@inp-greifswald.de (E.F.); woedtke@inp-greifswald.de (T.v.W.); 2 Department of General, Visceral, Thoracic and Vascular Surgery, Greifswald University Medical Center, Ferdinand-Sauerbruch-Str., 17475 Greifswald, Germany; 3 Institute for Hygiene and Environmental Medicine, Greifswald University Medical Center, Walther-Rathenau-Str. 48, 17489 Greifswald, Germany

**Keywords:** kINPen, MNT-1, oncology, plasma medicine, reactive oxygen species, ROS, SK-MEL-28

## Abstract

**Simple Summary:**

Despite recent advances in therapeutic options, melanoma remains a deadly disease with a poor prognosis. Physical gas plasma has been proposed as a promising technology for the treatment of melanoma. This study aimed to develop and investigate a convenient test system based on three-dimensional cell cultures (spheroids) of two melanoma cell lines in response to physical gas plasma. The experimental approach combined high-content imaging technology and different gas plasma treatment modalities (direct and indirect, gas compositions). Our results revealed that plasma treatment was toxic for both cell lines predominantly dependent on the treatment time. Furthermore, we addressed the question of safety and morphological changes in response to physical gas plasma exposure and found no support for metastatic progression. Treatment with physical gas plasma effectively limited the growth of human 3D melanoma spheroids and provided a versatile test system for more in vivo-like tumor tissue.

**Abstract:**

Melanoma skin cancer is still a deadly disease despite recent advances in therapy. Previous studies have suggested medical plasma technology as a promising modality for melanoma treatment. However, the efficacy of plasmas operated under different ambient air conditions and the comparison of direct and indirect plasma treatments are mostly unexplored for this tumor entity. Moreover, exactly how plasma treatment affects melanoma metastasis has still not been explained. Using 3D tumor spheroid models and high-content imaging technology, we addressed these questions by utilizing one metastatic and one non-metastatic human melanoma cell line targeted with an argon plasma jet. Plasma treatment was toxic in both cell lines. Modulating the oxygen and nitrogen ambient air composition (100/0, 75/25, 50/50, 25/75, and 0/100) gave similar toxicity and reduced the spheroid growth for all conditions. This was the case for both direct and indirect treatments, with the former showing a treatment time-dependent response while the latter resulted in cytotoxicity with the longest treatment time investigated. Live-cell imaging of in-gel cultured spheroids indicated that plasma treatment did not enhance metastasis, and flow cytometry showed a significant modulation of S100A4 but not in any of the five other metastasis-related markers (β-catenin, E-cadherin, LEF1, SLUG, and ZEB1) investigated.

## 1. Introduction

Skin cancer, including actinic keratosis, is the most common malignancy in humans, and malignant melanoma has the highest mortality rate. Melanoma represents more than 75% of skin cancer-related deaths [1]. Melanomas derive from cutaneous pigment-producing melanocytes that undergo malignant transformation [2]. Due to their close vicinity to the basement membrane and its aggressiveness, the invasion of the dermal layer occurs frequently and worsens the prognosis of patients due to the formation of metastasis elsewhere. Moreover, the high mortality rate in melanoma patients is due to the high mutation rate, which is frequently accompanied by therapy resistance [3]. Therefore, onco-dermatological research aims to find effective therapeutic approaches to tackle this disease. The development of effective treatment modalities for melanoma remains challenging, and fast and reproducible in vitro and preclinical test systems are needed. 3D spheroid cultures of melanoma cells offer a useful test system that bridges the gap between in vitro 2D cell culture and in vivo animal models [4,5]. In combination with methodological improvements in high-throughput manipulation and analysis methods, these 3D melanoma models are of interest for in vitro oncology studies. The benefit of 3D spheroids is that they better reflect the tumor microenvironment than tumor cells grown in monolayers (2D culture). However, 3D tumor spheroids lack vascularization and immune cell infiltration, which are important factors that affect tumor growth in vivo. Yet, and similar to tumors established in vivo, 3D spheroids show cellular heterogeneity and an oxygen gradient, which can form a hypoxic core, especially if they are larger than 500 µm in diameter [6,7]. In addition, the cell-cell interactions closely resemble those found in the tumor microenvironment (TME), which is composed of an extracellular matrix (ECM). The use of a cell-free matrigel provides an even more realistic setup for investigating 3D tumor spheroids with a particular focus on cell migration and invasion through the extracellular matrix [8,9]. Moreover, state-of-the-art high-content imaging allows for the in-depth dynamic monitoring of invading cells without compromising the embedded spheroids.

The risk factors for cutaneous melanoma include chronic sun exposure and increased age, but they can also appear in young children [10]. While the primary tumor lesions of non-metastatic tumors are mostly successfully targeted, leading to good 5-year survival rates, metastatic malignant melanoma is still associated with poor clinical outcomes [11]. Cells derived initially from primary or metastatic tumor sites retain a share of their in vivo phenotypes in vitro. For instance, the cell line SK-MEL-28 that was used in this current study and is derived from a primary tumor site shows a marked proliferative but not invasive signature [12]. The origin of the melanoma cell has also been found to be related to resistance to oxidative stress [13]. This type of cell stress is induced by an overload of reactive oxygen species (ROS) that can also perform signaling functions [14] and have been implicated in promoting metastasis [15]. On the contrary, it has also been suggested that ROS has a beneficial role in the therapy of cancer. These therapeutic ROS can be generated intracellularly by drugs [16] by using photodynamic therapy [17], and by using a recently established technology, cold physical plasma [18].

Over the past several years, evidence has accumulated suggesting that cold physical plasma or plasma-treated liquids can act as a promising agent for anticancer therapy. Either plasma-treated liquids (indirect) or direct plasma treatment were tested on a variety of cancer cells and test models [18,19,20]. Based on these studies, it emerged that ROS play a significant role in triggering a cascade leading to cell death in vitro [21,22]. A large number of promising studies have been published on the use of medical plasma technology to kill melanoma cells in vitro and in vivo [23]. In the field of plasma medical research, one goal is to define the specific biological response of different reactive species. Recently, we designed a shielding device for the plasma jet kINPen that provides a ROS-enriched plasma or an RNS-enriched plasma by fully shielding the plasma effluent from ambient air with pure oxygen or nitrogen or combinations of the two [24,25]. While the generated plasma species in the gas phase are well-studied [26,27,28,29], their cellular effects in cancer treatment still need to be evaluated. So far, it is not known whether a gas composition other than ambient air will have a more potent cytotoxic effect in cancer cells in general and in melanoma spheroids in particular. The aim of this study was, therefore, to investigate the influence of the ambient gas composition on plasma-mediated toxicity and growth in two cell lines that form 3D multicellular spheroids. Additionally, outgrowth and cell migration were tested by embedding spheroids into an extracellular matrix. By employing high-content imaging and sophisticated image analysis, we were able to quantitatively assess the effects of ambient gas composition, cell type, and the evasion properties of 3D melanoma spheroids following plasma treatment.

## 2. Results

### 2.1. Direct and Indirect Plasma Treatment Was Cytotoxic in Melanoma Spheroids

**Figure 1:**
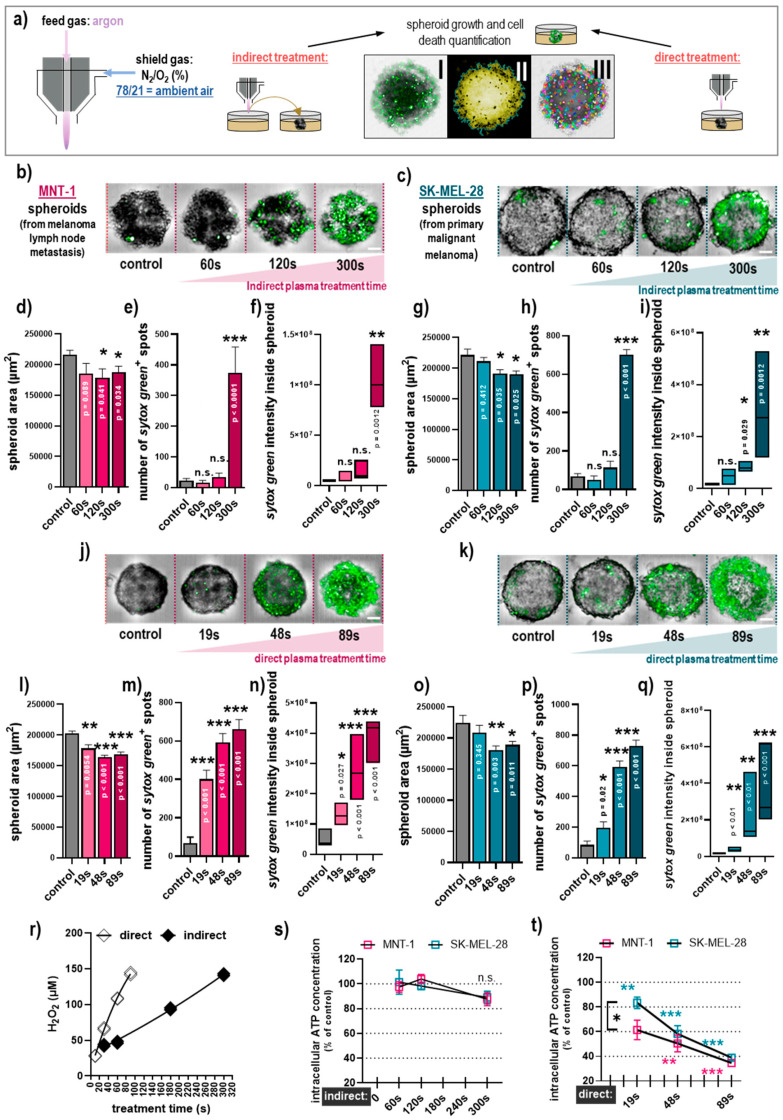
Comparison of direct and indirect plasma treatment under ambient air conditions in human 3D melanoma spheroids. (**a**) Schematic presentation of the treatment setup with I showing the brightfield and sytox green overlay image, II showing the spheroid area being segmented, and III showing the spot analysis segmented based on sytox green; (**b**,**c**) representative images of MNT-1 (**b**) and SK-MEL-28 (**c**) 3D spheroids following indirect plasma treatment; (**d**–**f**) quantification of spheroid area (**d**), number of sytox green^+^ spots (**e**), and sum intensity of sytox green (**f**) in MNT-1 cells following indirect plasma treatment; (**g**–**i**) quantification of spheroid area (**g**), number of sytox green^+^ spots (**h**), and sum intensity of sytox green (**i**) in SK-MEL-28 cells following indirect plasma treatment; (**j**,**k**) representative images of MNT-1 (**j**) and SK-MEL-28 (**k**) 3D spheroids following direct plasma treatment; (**l**–**n**) quantification of spheroid area (**l**), number of sytox green^+^ spots (**m**), and sum intensity of sytox green (**n**) in MNT-1 cells following direct plasma treatment; (**o**–**q**) quantification of spheroid area (**o**), number of sytox green^+^ spots (**p**), and sum intensity of sytox green (**q**) in SK-MEL-28 cells following direct plasma treatment; (**r**) concentration of hydrogen peroxide (H_2_O_2_) introduced via plasma treatment of cell culture medium alone in a 60mm culture dish (containing 5 mL of cell culture medium) or in a 96-well spheroid plate (containing 150 µL of cell culture medium); (**s**,**t**) quantification of ATP in indirectly (**s**) and directly (**t**) plasma-treated MNT-1 and SK-MEL-28 melanoma cells normalized to each untreated control. Data are mean +S.E. or boxplots of three independent experiments with three replicates each (except in s for MNT-1 with two independent experiments). Statistical analysis was performed using one-way analysis of variances comparing with Dunnett post hoc testing (**d**–**i**,**l**–**q**,**s**,**t**) or *t*-test (**s**,**t**) with *p* < 0.05 (*), *p* < 0.01 (**), and *p* < 0.001 (***). Scale bar = 100 µM; n.s. = not significant.

Using plasma treatment and two types of 3D melanoma spheroids, our study aimed to investigate the impact of (i) the ambient gas composition employed, (ii) the treatment regimen (direct vs. indirect) used, and (iii) the cell type (Figure 1a). Indirect plasma treatment under ambient air conditions was cytotoxic in MNT-1 (Figure 1b) and SK-MEL-28 (Figure 1c) tumor spheroids in a treatment time-dependent fashion. In contrast, in MNT-1 cells, the treatment resulted in a small but significant decrease in the spheroid size (Figure 1d) and the number of dead cells (Figure 1e), and the overall intensity of the dead cell marker sytox green (Figure 1f) was markedly elevated. Both the number of dead cells and the sum intensity of dead cells were obtained as sytox green also penetrates the early apoptotic cells to some extent. Finding a similar overall trend in both criteria suggests that there was terminal cell death within the spheroids. Similar results were found in SK-MEL-28 spheroids (Figure 1g–i), suggesting similar toxicity in both cell lines with this regimen. Notably, the amplitude of cell death was only elevated significantly for the most extended treatment time of 300 s, while the shorter treatment times were much less effective. Next, we measured the absolute hydrogen peroxide (H_2_O_2_) concentrations generated in the plasma-treated medium (indirect) in 5 mL of the 60 mm dishes and compared them to those generated in 150 µL of plasma-treated medium in wells of 96-well plates. This was done to find a concentration-matched plasma treatment time that generated equivalent amounts of hydrogen peroxide for the indirect (60 mm dish) and the direct (96-well plates) plasma treatment of tumor spheroids (Figure 1r). Similar to the indirect plasma treatment, toxicity was observed in a treatment time-dependent manner in MNT-1 (Figure 1j) and SK-MEL-28 (Figure 1k) tumor spheroids after direct plasma treatment. A reduction in the total spheroid area and an increase in the number of dead cells, as well as the sum intensity of the dead cell marker, was observed in both cell lines as well (Figure 1l–q). However, there was a clear difference between the treatments; in the direct treatment (but not in the indirect treatment) both long and short exposure times caused significant cell deaths. Moreover, analysis of ATP as a surrogate for cell activity and proliferation in lysed tumor spheroid material was performed for the indirect (Figure 1s) and direct (Figure 1t) plasma treatment. Indirect treatment did not produce a significant decrease in ATP levels in both cell types (Figure 1s). In contrast, direct plasma treatment had a stronger effect, and a small but significant difference between cytotoxicity in MNT-1 and SK-MEL-28 was found for the short direct plasma treatment times (Figure 1t). These data emphasized that the direct plasma treatment was vastly more toxic than the indirect regimen, especially for short and intermediate treatment times.

### 2.2. The Cytotoxicity of Direct and Indirect Plasma Treatment Was Independent of the Ambient Gas Composition Used

**Figure 2:**
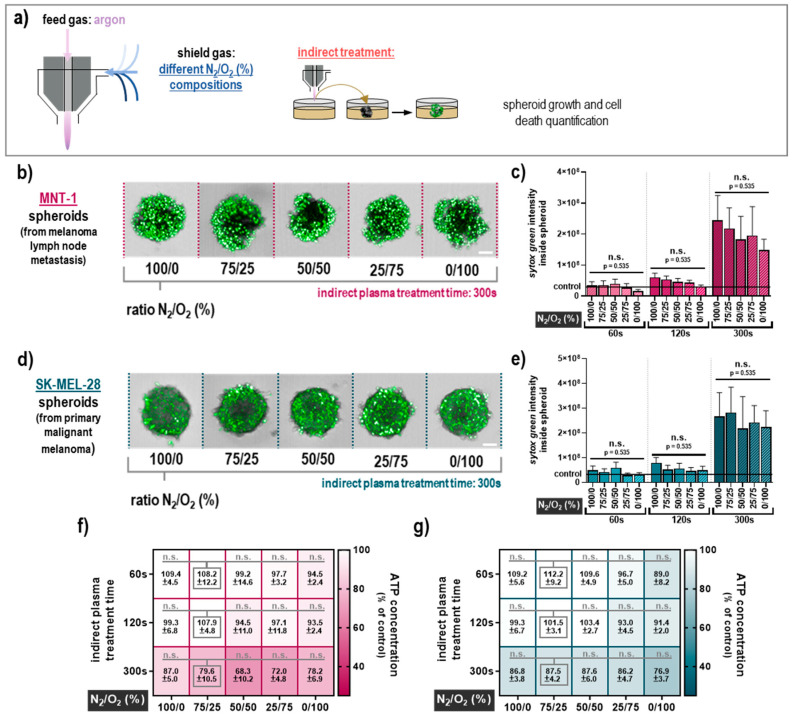
Cytotoxicity of indirect plasma treatment under different shield gas conditions. (**a**) Schematic presentation of the treatment setup with the plasma jet operated with argon and the shielding device filled with different N_2_/O_2_ compositions (ratios were 100/0, 75/25, 50/50, 25/75, and 0/100); (**b**) representative images of sytox green staining for all tested gas compositions after incubation in plasma-treated cell culture medium (300 s) for MNT-1 cells; (**c**) quantitative image analyses of sytox green intensity inside the MNT-1 spheroids; (**d**) representative images of sytox green staining for all tested gas compositions after incubation in plasma-treated cell culture medium (300 s) for SK-MEL-28 cells; (**e**) quantitative image analyses of sytox green intensity inside the SK-Mel-28 spheroids; (**f**,**g**) heat map for normalized ATP concentration (percent of controls) for all tested treatment conditions in MNT-1 (**f**) and SK-MEL-28 (**g**) spheroids. Data are mean +S.E. of three independent experiments with three replicates each. Statistical analysis was performed using two-way analysis of variances comparing the effects of each treatment time and feed gas composition with Dunnett posthoc testing against the respective 75/25 (N_2_/O_2_) feed gas composition as a surrogate for ambient air. Scale bar = 100 µM; n.s. = not significant.

**Figure 3:**
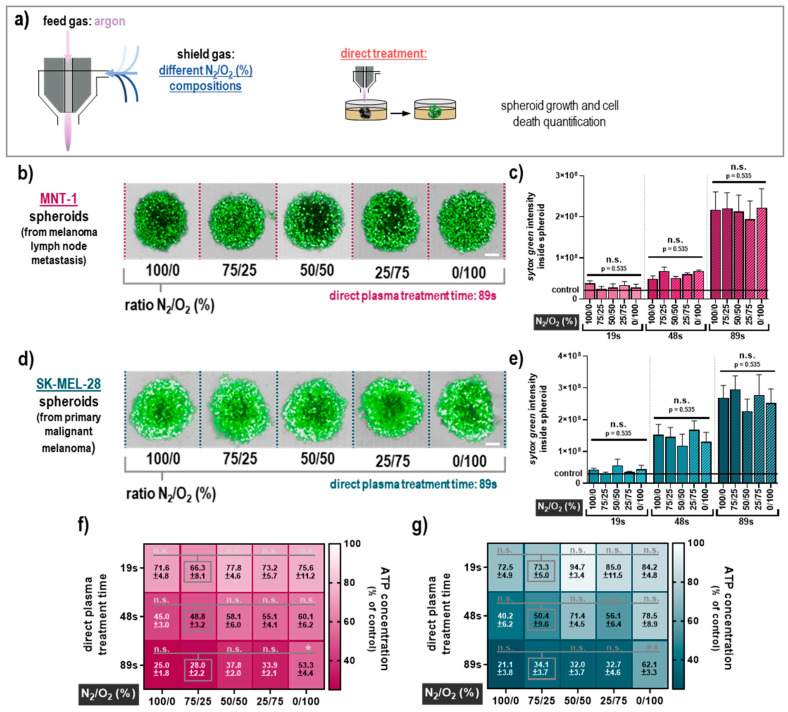
Cytotoxicity of direct plasma treatment under different shield gas conditions. (**a**) Schematic presentation of the treatment setup with the plasma jet operated with argon and the shielding device filled with different N_2_/O_2_ compositions (ratios were 100/0, 75/25, 50/50, 25/75, and 0/100); (**b**) representative images of sytox green staining for all tested gas compositions after direct plasma treatment (89 s) for MNT-1 cells; (**c**) quantitative image analyses of sytox green intensity inside the MNT-1 spheroids; (**d**) representative images of sytox green staining for all tested gas compositions after direct plasma treatment (89 s) for SK-MEL-28 cells; (**e**) quantitative image analyses of sytox green intensity inside the SK-MEL-28 spheroids; (**f**,**g**) heat map for normalized ATP concentration (percent of controls) for all tested treatment conditions in MNT-1 (**f**) and SK-MEL-28 (**g**) spheroids. Data are mean +S.E. of three independent experiments with three replicates each. Statistical analysis was performed using two-way analysis of variances comparing the effects of each treatment time and feed gas composition with Dunnett posthoc testing against the respective 75/25 (N_2_/O_2_) feed gas composition as a surrogate for ambient air with *p* < 0.05 (*) and *p* < 0.01 (**). Scale bar = 100 µM; n.s. = not significant.

The next question was whether the composition of the air surrounding the plasma effluent, which is known to be essential for the profile of the reactive species being generated, affects the amplitude of the cytotoxicity in 3D melanoma spheroids. To control the ambient air composition, a self-designed shielding gas device was employed to independently control the gas composition in the immediate vicinity to the plasma (Figure 2a). Five different nitrogen (N_2_) to oxygen (O_2_) ratios were tested (100/0, 75/25, 50/50, 25/75, and 0/100) for modulating the RNS and ROS chemistry in the plasma effluent. For the indirect plasma treatment of MNT-1 spheroids (Figure 2b), the different shielding gas compositions were similar in cytotoxicity (Figure 2c), and the oxygen concentrations generally correlated with a decrease in the plasma-induced cell death. In SK-MEL-28 spheroids (Figure 2d), this trend was less pronounced, while the surrounding gas composition did not have a significant effect on the plasma-mediated toxicity (Figure 2e). The variation in the ratio of O_2_ and N_2_ to the plasma shielding gas had little effect in MNT-1 (Figure 2f) and SK-MEL-28 (Figure 2g) in terms of ATP concentration, which is in good agreement with the sytox green staining. Similar results were achieved for the direct plasma treatment (Figure 3a) in MNT-1 cells (Figure 3b) concerning the modulation of shielding gas (Figure 3c). Data for the SK-MEL-28 cells (Figure 3d) were not much different (Figure 3e). Observations regarding the ATP data from MNT-1 (Figure 3f) and SK-MEL-28 (Figure 3g) were in line with these findings, but they also highlighted the tendency for cytotoxicity to be impaired under the 100% oxygen (absence of nitrogen) condition compared to the other shield gas settings. This tendency was also significant in MNT-1 (Figure 3f) and SK-MEL-28 cells (Figure 3g) for the longest direct treatment time. This finding, however, was only apparent in the ATP assay but not in the imaging results. Altogether, these data suggest that the impact of the nitrogen-to-oxygen ratio near the plasma effluent is negligible in terms of tumor-toxic effects in 3D multicellular human melanoma spheroids with both direct and indirect plasma treatment regimens. One exception was a significantly reduced cytotoxicity for long direct plasma treatment times in the absence of nitrogen.

### 2.3. Plasma Treatment Impaired Migration of Melanoma Cells out of the Spheroid

**Figure 4:**
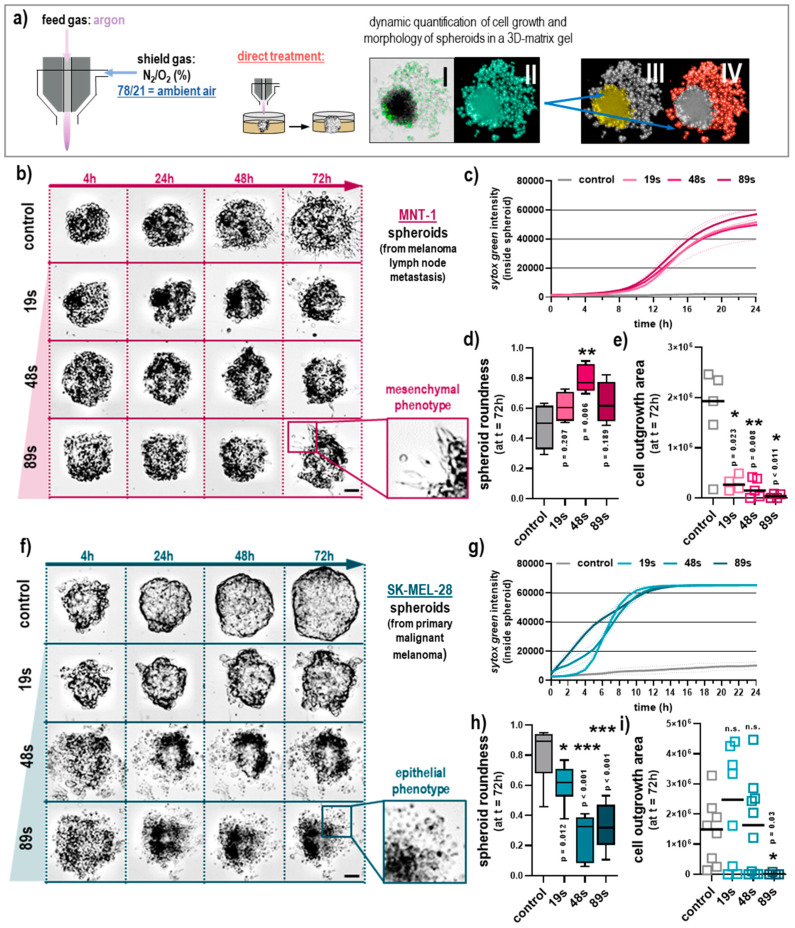
Quantification of evasion properties in plasma-treated melanoma spheroids under ambient air conditions in a 3D-matrix gel. (**a**) Schematic presentation of the workflow and the high-content image analysis showing the brightfield and sytox green overlay image (I), the total area segmented from that image (II), the spheroid core segmentation (III), and the segmented region containing cells outside of the spheroid core (IV); (**b**) representative images of plasma-treated MNT-1 spheroids; (**c**) live-cell imaging of MNT-1 spheroids over 24 h and quantification of the cell death marker kinetics; (**d**) morphological analysis of MNT-1 tumor spheroids at 72 h; (**e**) quantification of viable MNT-1 cells growing outside the core tumor spheroid at 72 h; (**f**) representative images of plasma-treated SK-MEL-28 spheroids; (**g**) live-cell imaging of SK-MEL-28 spheroids over 24 h and quantification of the cell death marker kinetics; (**h**) morphological analysis of SK-MEL-28 tumor spheroids at 72 h; (**i**) quantification of viable SK-MEL-28 cells growing outside the core tumor spheroid at 72 h. Data are mean ±S.E., boxplots, or mean with individual values and of two independent experiments with four replicates each, the sytox green kinetics show the mean (solid lines) and SD (dashed lines). Statistical analysis was performed using one-way analysis of variances comparing with Dunnett posthoc testing with *p* < 0.05 (*), *p* < 0.01 (**), and *p* < 0.001 (***). Scale bar = 100 µM; n.s. = not significant.

The results above revealed only minor differences between the different shielding gas mixtures, while the direct plasma treatment showed elevated toxicity. Since cell culture medium cannot be applied in clinics, the direct plasma treatment was used in the subsequent experiments. Similarly, since none of the shielding gas mixtures were particularly effective, we subsequently omitted the shielding gas and used the ambient air in a putative clinical application. With 78% N_2_ and 21% O_2_, ambient air closely resembles the 75/25 N_2_/O_2_ condition mentioned earlier in the study. For the following experiments, the spheroids of SK-MEL-28 and MNT-1 melanoma cells were directly exposed to a plasma effluent running at ambient air condition (Figure 4a). The interaction of the melanoma tumor spheroid and its cells with the extracellular matrix (ECM) was simulated by embedding the plasma-treated spheroids into matrigel that supports migration and invasion. In an attempt to investigate cellular migration behavior upon plasma treatment, spheroids were fully submerged in matrigel and cultivated for 72 h. Images were captured after 4 h, 24 h, 48 h, and 72 h using high-content imaging. Brightfield images overlaid with fluorescence images allowed the segmentation of the spheroid core morphology and the quantification of cells outside the spheroid. The melanoma cells were labeled with the fluorescent tag DiL added before spheroid formation. Data analysis came from an algorithm-driven image quantification with no manual intervention. After 72 h, MNT-1 spheroids showed some migrating cells in the untreated control and after plasma exposure for all tested treatment times (Figure 4b). Live-cell imaging for cell death kinetics showed an onset of toxicity in MNT-1 cells at about 12 h following plasma treatment (Figure 4c). At 72 h after plasma treatment, the spheroids of MNT-1 cells remained round although some spheroids lost their compactness and the structure was resolved, and occasionally spheroids showed an altered morphology (Figure 4d) for longer treatment times. Moreover, plasma treatment reduced the number of metastatic cells evading the spheroid core into the surrounding matrix (Figure 4e). MNT-1 cells are invasive and highly metastatic (mesenchymal) because they are derived from a lymph node metastasis, while SK-MEL-28 cells are derived from a primary melanoma tumor with a less pronounced metastatic phenotype (epithelial) in the controls (Figure 4f). Live-cell imaging of the cell death kinetics showed an onset of toxicity in SK-MEL-28 cells as early as 2 h after plasma treatment (Figure 4g). Unlike MNT-1 cells, plasma-treated SK-MEL-28 spheroids lost their round shape and compactness, especially for longer plasma exposure times (Figure 4h). Individual cells outside the main spheroid were found, as demonstrated in the images shown in Figure 4f. These cells, however, did not actively migrate outside the spheroid but rather were leftover cells from the initial spheroid falling apart. This can be observed in the image in Figure 4a, which shows that most of the cells were sytox green^+^ (dead) with the round morphology of evading cells, which points to a non-metastatic, epithelial phenotype (Figure 4f). Accordingly, the quantitative image analysis of viable cells outside the tumor spheroid region showed mostly similar numbers of evading cells (Figure 4i) in the plasma conditions. These data were supported by continuous live-cell imaging of control and plasma-treated spheroids in matrigel for MNT-1 cells over 24 h (Supplementary Materials, Video S1). The videos show that cells within the spheroids are still alive after plasma treatment but fail to exit the spheroid. In contrast, untreated MNT-1 spheroids in gel show extensive motility of individual cells outside the central spheroid region. These data strongly suggest that plasma treatment reduces not only spheroid viability but also metastatic spread.

### 2.4. Epithelial-Mesenchymal-Transition (EMT) Markers after Plasma Treatment in Human Melanoma Spheroids

**Figure 5:**
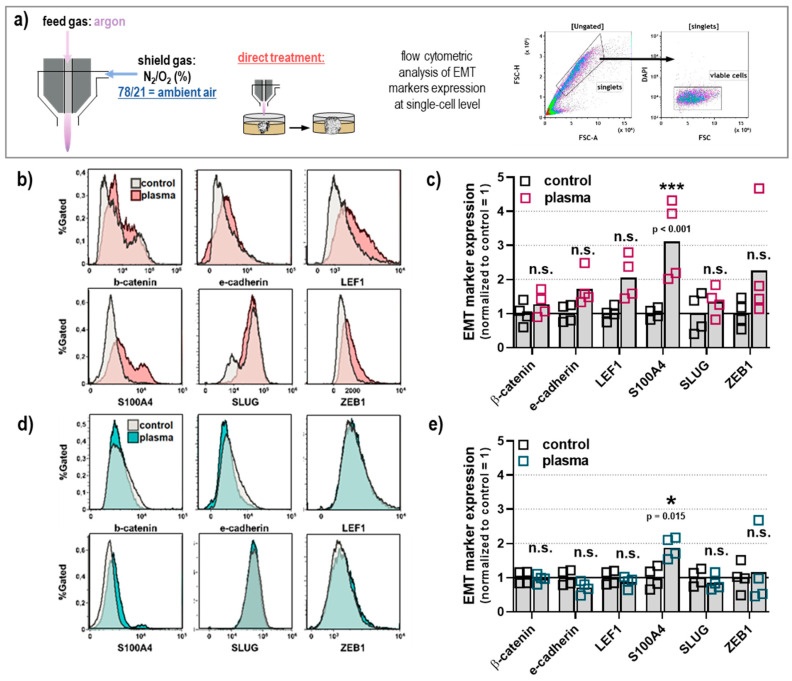
Plasma treatment under ambient conditions of melanoma spheroids had a minor effect on EMT marker expression. (**a**) Schematic presentation of the plasma treatment (89 s) of spheroids embedded in matrigel, and subsequent flow cytometric analysis of the EMT markers β-catenin, E-cadherin, LEF-1, S100A4, SLUG, and ZEB1 at the single-cell level from dissociated spheroids; (**b**) representative fluorescence overlay histograms from flow cytometry of MNT-1 cells; (**c**) quantitative analysis of flow cytometry data from MNT-1 cells; (**d**) representative fluorescence overlay histograms from flow cytometry of SK-MEL-28 cells; (**e**) quantitative analysis of flow cytometry data from SK-MEL-28 cells. Data are mean with individual values of two independent experiments with two replicates each. Statistical analysis was performed using two-way analysis of variances with Sidak post hoc testing and with *p* < 0.05 (*) and *p* < 0.001 (***). n.s. = not significant.

Plasma treatment decreased the area of outgrowth in MNT-1 metastatic melanoma spheroids while impairing the spheroid and cellular integrity of non-metastatic SK-MEL-28 melanoma cells, which demonstrated that plasma treatment inhibits spheroid-based migration of individual cells in the ECM matrigel. Phenotypically, SK-MEL-28 represents the epithelial phenotype, whereas MNT-1 cells resemble the mesenchymal phenotype, which are visible as round-shaped cells and elongated cell shape, respectively. Distinct cell surface markers are generally used as indicators for the EMT in cancer cells [30]. In this study, we used selected markers in individual cells from dissociated SK-MEL-28 and MNT-1 spheroids after plasma treatment to investigate their metastatic capacity further (Figure 5a). To do so, the spheroids were embedded in matrigel as before, followed by enzymatic dissociation before FACS analyses for the EMT marker β-catenin, E-cadherin, LEF1, S100A4, SLUG, and ZEB1 (Figure 5b). The plasma exposure and ECM interaction resulted in a significantly increased expression of S100A4 in MNT-1 cells (Figure 5c), while the expression of all other markers investigated was not significantly enhanced. The analysis of the EMT marker in SK-MEL-28 cells (Figure 5d) showed only a small but significant increase of S100A4, while the expression of all other markers was not significantly changed (Figure 5e).

## 3. Discussion

The first studies on the use of cold physical plasma for the elimination of melanoma cells were conducted more than a decade ago [31]. Since then, the use of cultured cells alone as a model system has been extended to more sophisticated model systems developed to investigate cold physical plasma as a treatment option [23]. Both in vitro and animal studies using cold atmospheric plasma for the treatment of melanoma have revealed promising results and significantly decreased the tumor growth or volume. Although not as complex as an entire organism, the use of spheroids offers a suitable 3D model that represents the cellular organization and regionalization of microtumors with nutrient and oxygen gradients [7]. In this study, we used metastatic and non-metastatic melanoma cells grown in 3D tumor spheroids to elucidate the effects of cold physical plasma following different application modes by employing high-content imaging technology.

To date, there is ample evidence that plasma-derived ROS and RNS play a significant role in inducing a cascade that eventually leads to cell death and tumor volume reduction [16,32,33]. In this work, we experimentally studied the impact of the argon-driven plasma jet kINPen on human melanoma spheroids in relation to the composition of the surrounding gas in the immediate vicinity to the plasma jet, which leads to a modulation of the composition of the reactive species that are eventually transported to the liquids and cells [25,34]. The viability decreased only in correlation to the treatment time but was not correlated to the reactive species composition except for the 100% O_2_ condition in the direct treatment regimen for long plasma exposure times. This could be explained by earlier findings that identified unchanged H_2_O_2_ concentration in biologically relevant liquids despite changes in reactive species patterns in the plasma gas phase following different ambient air conditions [35]. Notably, similar results were achieved for the inactivation of bacteria using the kINPen equipped with a shielding device in the conditions void of either O_2_ or N_2_ [36]. Previously, it was also found that the kINPen toxicity in human HaCaT keratinocytes was the highest in N_2_-free plasma conditions [37]. Such conditions still produced vast amounts of hydrogen peroxide but not nitrite and nitrate in liquids [36], which is indicative of an under-represented RNS chemistry that involves, for instance, nitric oxide and peroxynitrite from the kINPen [37,38]. Such chemistry seems necessary for maximizing cytotoxic effects in prokaryotes and eukaryotes apart from hydrogen peroxide and hypochlorous acid, which have often been found to be essential for plasma-induced cell death in liquid-rich culture systems [39,40,41]. A limitation of our study was that only the most extended treatment times were matched via hydrogen peroxide, while the shorter treatment times for the indirect treatment were one third and one fifth, and the direct treatment was one half and one-fourth of the maximum treatment time. Although this limits any direct comparison to some extent, the more substantial toxicity of the longest direct treatment time compared to the longest indirect time still suggests that the direct plasma treatment is more potent than the indirect treatment.

Among cutaneous melanocytes, the synthesizing capacity of melanin differs between skin phototypes, which correlate with the content of melanin and skin color [42]. Melanin is a biopolymer derived from the oxidation of the aromatic amino acids L-phenylalanine and L-tyrosine and is the main characteristic of melanocytes. While melanin protects normal melanocytes from oxidative stress and UV radiation [43], it also chelates metal cations, consumes intracellular oxygen (causing hypoxia), and can contribute to therapy resistance of melanoma cells against, for instance, chemotherapy and radiotherapy [44]. Although amelanotic melanomas are rare and account for only 1-8% of all melanomas, the prognosis is as bad as for pigmented melanomas, and the treatment follows the same process [45]. Although photodynamic therapy can be used for the treatment of melanoma, the outcome is negatively influenced by high pigmentation [46]. Using amelanotic SK-MEL-28 and highly pigmented MNT-1 cells allowed us to estimate whether the melanin content affected the outcome of the plasma application in vitro. Both SK-MEL-28 and MNT-1 responded to cytotoxic plasma treatment to a similar extent, and inhibited the role of melanin in plasma-induced cell death in our model. However, this preliminary conclusion must be supported by the use of more cell lines and inhibitor studies in the future. Subtle differences in the ATP content were observed between plasma-treated SK-MEL-28 and MNT-1 cells that may relate to differences in cell proliferation and hence the number of cells producing ATP rather than the immediate occurrence of cell death. An apparent difference between both cell lines, however, is their origin, with SK-MEL-28 being derived from a primary lesion and MNT-1 from a metastatic lesion. However, we found that plasma treatment decreased cellular outgrowth in both cell types, indicating plasma exposure to therapeutically targeted cell types to a similar extent. Yet, our preliminary conclusion on the role of melanin content and epithelial or mesenchymal phenotype related to plasma treatment should be investigated further by the use of more cell lines and inhibitor studies in the future. We were also unable to use primary melanocytes or HaCaT keratinocytes as controls in our study as these cell lines did not form multicellular spheroids. Nevertheless, the similar responses observed for SK-MEL-28 and MNT-1 might be related to the findings from gene expression data from publicly available resources that suggest that both cell types have overall similar expression patterns.

Our results imply that using direct plasma treatment of melanoma lesions with the kINPen MED in ambient air has the greatest chance of therapeutic success. In good agreement with a recent report [20], we found that direct plasma exposure in two melanoma cell lines was more effective in reducing the spheroids’ cell viability than indirect treatments. Our previous work investigated the use of plasma-conditioned liquids as a therapeutic strategy to combat tumors of the peritoneum [47,48,49]. In our study, we compared both treatment options, direct plasma exposure and exposure to plasma-treated liquid. While the first option delivers short-lived ROS and RNS to the biological target, the second contains mostly long-living ROS and RNS, such as hydrogen peroxide, nitrite, and nitrate [50]. To compare the impact of both regimens, the treatment times were adjusted according to the hydrogen peroxide concentration, and direct treatment was more toxic than indirect treatment. This was somewhat unexpected since the indirect treatment accumulates large amounts of ROS that are added to the cells at once, while the direct treatment continuously transfers ROS to the target. The latter situation allows for some ROS to react with or to be scavenged by organic molecules, and preventing the high build-up of concentrations, as was found in the indirect treatment [51]. The indirect treatment is void of short-lived reactive species that are known to be toxic at higher concentrations, such as peroxynitrite and singlet oxygen [52,53].

The ECM of basement membrane extracts, known as matrigel, offers an environment that supports ECM-tumor-cell interaction and the study of migration, invasion, and therapeutic responses [9]. The addition of an ECM to the melanoma spheroid represents a more in vivo-like tumor tissue construct than conventional 2D culture and 3D spheroids in the cell culture medium. Real-time imaging revealed a spatial distribution of dead cells that decrease from the outside to the inside of the spheroids that is most obvious for shorter treatment times. We also subsequently dissociated the spheroids for further FACS analyses in order to determine EMT marker expression. Metastatic melanoma cells revealed a large increase in the expression of S100A4 following plasma treatment, and non-metastatic melanoma cells showed an increase to a minor extent. At the same time, plasma treatment decreased the spheroid area and increased the number of dead cells outside the core spheroid. Moreover, although the plasma treatment resulted in some spheroid loosening, as indicated by the change in the roundness of the core spheroid, these cells did not migrate into the surrounding matrigel. This suggests that despite increased S100A4 expression, tumor metastasis was de facto not enhanced but even decreased with plasma treatment as the motility data show. The increased S100A4 expression might also be because the plasma treatment killed many of the melanoma cells. It might be assumed that this killing process selected for clonogenic variants that not only withstand ROS-induced cell death to a greater extent but also have intrinsically higher expression levels of S100A4.

A limitation of the direct treatment that has yet to be overcome is that most skin melanomas are located beneath the stratum corneum, which plasma-generated species are unlikely to pass, not to mention hard-to-reach metastasis inside of organs. Based on this, the kINPen plasma treatment might be an option for the therapy of ulcerating melanoma lesions in the palliative setting. Successful proof-of-concept studies on this approach have already been published for the palliative treatment of patients suffering from head and neck cancer [54,55]. Moreover, apoptosis was found in head and neck cancer and malignant melanoma lesions of patients after plasma treatment ex vivo [56,57]. This is supported by results in melanoma-bearing mice that showed reduced tumor growth following exposure to cold physical plasma [58,59]. Also, in vitro, several studies have reported the selective activity of plasma treatment against malignant over non-malignant cells [60,61]. At least for the kINPen plasma jet, evidence points to its safe use [62], which has paved the way for its accreditation as a medical product in the European Union. Specifically, it was shown that plasma treatment does not promote the formation of micronuclei (as per OECD assay guidelines) [63,64,65], and mice receiving several plasma treatments did not have long-term effects and cancerogenesis one year after plasma exposure [66]. Similar results were found in human volunteers [67,68].

## 4. Materials and Methods

### 4.1. Cell Culture and Spheroid Formation

The human melanoma cell line SK-MEL-28 (ATCC HTB-72) is derived from a skin melanoma, and MNT-1 (ATCC CRL-3450) is derived from a metastatic lymph node were employed in this study. Both cell lines were authenticated independently (Eurofins Genomics, Ebersberg, Germany). Cross-contamination was regularly precluded during the experiments. They also differ in their endogenous melanin content, with SK-MEL-28 being amelanotic and MNT-1 being highly pigmented. Despite those differences, both cell lines have comparable gene expression profiles. Both cell lines were maintained in RPMI1640 cell culture medium supplemented with 2 mM L-glutamine, 100 IU/mL penicillin, 100 µg/mL streptomycin (all PAN-Biotech, Aidenbach, Germany), and 8% fetal calf serum (Sigma-Aldrich, Steinheim, Germany). Cells were cultured in a humidified atmosphere at 37 °C and 5% CO_2_ in tissue culture flasks. The medium was changed every other day. Cells were harvested by the addition of 3 mL of 0.05% trypsin and 0.53 mM EDTA (Corning, Wiesbaden, Germany) before spheroid formation. Spheroid formation was induced by transferring a cell suspension (500 MNT-1 and 1000 SK-MEL-28 in 50 µL of cell culture medium) into a 96-well ultra-low attachment (ULA) plate with a round bottom (Nunclon Sphera Microplates; Thermo Scientific, Dreieich, Germany). The cell numbers were determined in pilot experiments that aimed to induce the formation of spheroids at about 500 µm in diameter on day 4. The plates subsequently underwent centrifugation at 1000× *g* for 10 min. Cell culture medium (100 µL) was added per well, and the plates were maintained in an incubator at 37 °C in a 5% CO_2_ humidified atmosphere. A portion of the culture medium (100 µL) was renewed every other day.

### 4.2. Plasma Jet and Treatment of Cells

The plasma jet kINPen MED (neoplas tools, Greifswald, Germany) was used to generate plasma at room temperature and ambient conditions. Argon (purity 99.999%; Air Liquide, Düsseldorf, Germany) was used as the carrier gas, which was ignited by applying a high voltage of 2–3 kV and a frequency of 1 MHz. During plasma generation, a pulsed mode with a frequency of 2.5 kHz was applied. The gas flow was set to three standard liters per minute. In order to modify the ambient conditions, a shielding device made of glass was employed. This way, the plasma effluent was well separated from the surrounding room air. The shielding gas consisted of different ratios of nitrogen and oxygen, starting from 0% O_2_ and 100% N_2_ going up in 5 steps to 100% O_2_ and 0% N_2_. In this study, the plasma was applied either directly to the cultured spheroids in the 96-well plate (direct treatment) or to cell culture medium that was then added to the wells of the 96-well plate containing 3D melanoma spheroids (indirect treatment mode). After plasma treatment, the medium was not changed. The particular treatment modality is shown schematically on the top of each figure. To quantify the amount of hydrogen peroxide (H_2_O_2_) that was introduced into the liquids, the Amplex Ultra Red Assay (Thermo Scientific, Waltham, MA, USA) was utilized as previously described [34].

### 4.3. ATP Assay

Cell viability was determined 24 h after plasma exposure using the CellTiter-Glo 3D Assay (Promega, Mannheim, Germany) according to the manufacturer’s instruction. The assay is based on ATP quantification as a marker for the presence of metabolically active cells. Briefly, spheroids were lysed in 50 µL of Cell Titer Glow Reagent and further diluted 1:10. The luminescence signal was recorded using a plate reader (M200; Tecan, Männedorf, Switzerland).

### 4.4. High-Content Imaging

Microscopy analysis of spheroids was done using an Operetta CLS high-content imaging device (PerkinElmer, Hamburg, Germany) equipped with laser-based autofocus. Spheroids were imaged using Z-stacks to generate maximum intensity projections using Harmony 4.9 quantitative imaging analysis software (PerkinElmer, Hamburg, Germany). The software was also used to segment regions of the spheroid, such as the core and outgrowth area, using image algorithms only. Fluorescence of sytox green (final concentration of 1 µM incubated for 15 min before imaging; ThermoFisher, Dreieich, Germany) was captured using appropriate excitation and emission wavelengths. For live-cell imaging over 24 h, the device was heated to 37 °C, and 5% CO_2_ was supplied. Evaporation protection was achieved by filling the outer wells of the 96-well plate with double-distilled water.

### 4.5. Migration of Melanoma Cells in a 3D-Matrix Gel

The melanoma cells were stained with Vybrant DiL (Thermo Scientific, Dreieich, Germany) for 15 min at 37 °C before spheroid formation was initiated as described above. Plasma treatment was performed on day four, followed by incubation for 4 h. Spheroids (with residual amounts of original medium) were transferred to a fresh round-bottom multiwell plate without adding new culture medium and overlaid with 50 µL of matrigel (Corning, Wiesbaden, Germany). Plates were centrifuged at 500× *g* for 5 min in a precooled centrifuge (4 °C). Matrigel was allowed to polymerize for 1 h at 37 °C, and 100 µL of culture medium was added. Spheroids and outgrowths of single melanoma cells were visualized in real-time using high-content imaging for 72 h. Fresh culture medium (50 µL) per well was added on day 2.

### 4.6. Flow Cytometry

A single-cell suspension was obtained by dissociation of the matrigel-spheroid-complex by adding 100 µL/well dispase II (1 mg/mL; Sigma-Aldrich, Steinheim, Germany) and subsequent incubation at 37 °C for 1 h. After the incubation, the dispase activity was stopped by the addition of 50 µL of PBS containing 5mM EDTA. The resulting cell suspension was fixed in 4% paraformaldehyde for 10 min, washed, and the technical replicates were pooled. A master mix (50 µL) containing fluorescently labeled antibodies targeting β-catenin (BD Pharmingen, Heidelberg, Germany), E-cadherin (CD324; BioLegend, London, UK), lymphoid enhancer-binding factor (LEF1; Cell Signaling, Frankfurt, Germany), S100A4, snail family zinc finger 2 (SLUG; SantaCruz, Heidelberg, Germany) as well as Zinc Finger E Box-Binding Homeobox 1 (ZEB1; R&D Systems, Wiesbaden, Germany) and Triton X-100 was added. After incubation for 20 min in the dark, the cell suspension was centrifuged and washed twice. FACS analyses were performed using a CytoFLEX S flow cytometer and Kaluza analysis 2.1 software (both Beckman-Coulter, Krefeld, Germany). To quantify the marker expression of both cell types, the geometric mean was quantified in the different channels on DAPI^-^ cells and was then normalized to the corresponding untreated controls.

### 4.7. Statistical Analysis

Statistical analysis was done using prism 8.4 (GraphPad Software, San Diego, CA, USA). The types of statistical analysis are given in the figure legends.

## 5. Conclusions

Plasma treatment effectively limited the growth of human 3D melanoma spheroids. For prolonged treatment times, this was independent of using a direct or indirect treatment regimen. For shorter treatment times, the direct treatment regimen was superior to indirect treatment. The cytotoxicity of direct and indirect plasma treatment was independent of the nitrogen-to-oxygen ratio in the surrounding gas of the plasma effluent except for oxygen-only-conditions, which was superior for direct treatment and long treatment times. Finally, plasma treatment did not promote tumor metastasis in human melanoma spheroids. For further analysis, future studies are needed that are dedicated to investigating the metastatic potential of plasma-treated melanoma lesions in vivo.

## Supplementary Materials

The following are available online at https://www.mdpi.com/2072-6694/12/9/2570/s1, Video S1: Live-cell imaging of untreated and plasma-treated MNT-1 spheroids in matrigel over 24 h imaged at an frequency of 1/h.
